# Two Autism/Dyslexia Linked Variations of *DOCK4* Disrupt the Gene Function on Rac1/Rap1 Activation, Neurite Outgrowth, and Synapse Development

**DOI:** 10.3389/fncel.2019.00577

**Published:** 2020-01-15

**Authors:** Miaoqi Huang, Chunmei Liang, Shengnan Li, Jifeng Zhang, Daji Guo, Bo Zhao, Yuyang Liu, Yinghui Peng, Junyu Xu, Wei Liu, Guoqing Guo, Lei Shi

**Affiliations:** ^1^JNU-HKUST Joint Laboratory for Neuroscience and Innovative Drug Research, College of Pharmacy, Jinan University, Guangzhou, China; ^2^Department of Anatomy, Medical College of Jinan University, Guangzhou, China; ^3^Shenzhen Key Laboratory for Neuronal Structural Biology, Biomedical Research Institute, Shenzhen Peking University – The Hong Kong University of Science and Technology Medical Center, Shenzhen, China; ^4^Department of Neurobiology, Key Laboratory of Medical Neurobiology of the Ministry of Health of China, Collaborative Innovation Center for Brain Science, Zhejiang University School of Medicine, Hangzhou, China

**Keywords:** autism, dendritic spine, Dock4, dyslexia, neuron, neurite, Rac1, Rap1

## Abstract

Autism spectrum disorder (ASD) and dyslexia are both neurodevelopmental disorders with high prevalence in children. Both disorders have strong genetic basis, and share similar social communication deficits co-occurring with impairments of reading or language. However, whether these two disorders share common genetic risks remain elusive. *DOCK4* (dedicator for cytokinesis 4), a guanine nucleotide exchange factor (GEF) for the small GTPase Rac1, is one of few genes that are associated with both ASD and dyslexia. Dock4 is important for neuronal development and social behaviors. Two *DOCK4* variations, Exon27-52 deletion (protein product: Dock4-945VS) and a missense mutation at rs2074130 (protein product: Dock4-R853H), are associated with dyslexia and/or ASD with reading difficulties. The present study explores the molecular and cellular functions of these two *DOCK4* variants on neuronal development, by comparing them with the wild-type Dock4 protein. Notably, it is revealed that both mutants of Dock4 showed decreased ability to activate not only Rac1, but also another small GTPase Rap1. Consistently, both mutants were dysfunctional for regulation of cell morphology and cytoskeleton. Using Neuro-2a cells and hippocampus neurons as models, we found that both mutants had compromised function in promoting neurite outgrowth and dendritic spine formation. Electrophysiological recordings further showed that R853H partially lost the ability to promote excitatory synaptic transmission, whereas 945VS totally lost the ability. Together, we identified R853 as a previously uncharacterized site for the regulation of the integrity of Dock4 function, and provides insights in understanding the common molecular pathophysiology of ASD and dyslexia.

## Introduction

Dock (dedicator of cytokinesis) protein family, a subtype of atypical guanine nucleotide exchange factors (GEFs) for promoting the activity of Rac1/Cdc42, has been implicated in regulating various processes of brain development and is linked with neurological diseases (Shi, 2013). Among them, Dock4 has attracted recent attention as emerging evidence suggests *DOCK4* as a candidate gene for several neuropsychiatric diseases, including autism spectrum disorder (ASD), dyslexia and schizophrenia (Maestrini et al., 2010; Pagnamenta et al., 2010; Poelmans et al., 2011; Alkelai et al., 2012; Iossifov et al., 2014; Liang et al., 2014; Toma et al., 2014; Warrier et al., 2015; Shao et al., 2016; Lim et al., 2017; Akahoshi and Yamamoto, 2018; Kushima et al., 2018). Our previous study using *Dock4* knockout mice have revealed that Dock4 deficiency *in vivo* leads to autism-like behaviors, including defects in social novelty preference and communication (Guo et al., 2019). In particular, impairment of Dock4-dependent excitatory synapse transmission in hippocampal CA1 pyramidal neurons is a main cause for the social deficits (Guo et al., 2019). Moreover, Dock4 is suggested to play important roles in neuronal development such as axon guidance, dendrite development and dendritic spine morphogenesis (Ueda et al., 2008, 2013; Xiao et al., 2013; Makihara et al., 2018). Dock4 possesses an N-terminal SH3 (Src-homology 3) domain, a DHR1 (Dock homology region 1) domain, and a DHR2 domain which is well-studied as a Rac1-specific GEF domain. Indeed, Rac1 was demonstrated to be a key molecule that mediates Dock4’s function in the above studies. Interestingly, evidence from other system suggests that Dock4 is also capable of activating Rap1 (Yajnik et al., 2003), another small G protein involved in cell adhesion and growth. This Rap1-activating function seems to be unique for Dock4, which has been the only Dock reported to possess this ability. However, whether Dock4 regulates Rap1 in the nervous system has not been studied.

**FIGURE 1:**
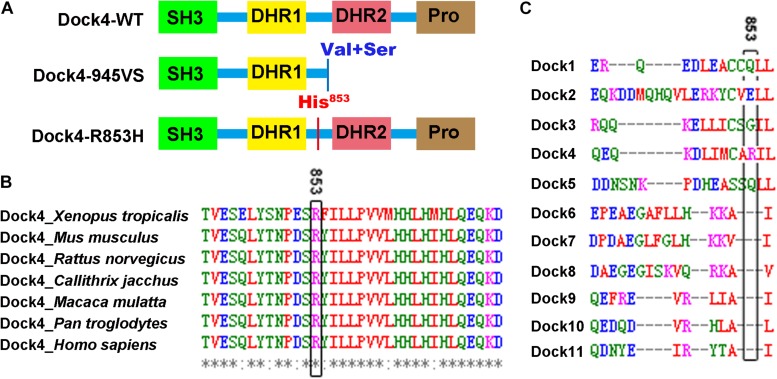
Arg^853^ is a unique site of Dock4 among all Dock family members and is highly conserved during evolution. **(A)** Domain illustrations of Dock4 mutants associated with autism spectrum disorder and dyslexia. **(B)** The 853^th^ Arginine residue of Dock4 is highly conserved in different species during evolution. **(C)** The 853^th^ Arginine residue of Dock4 is not conserved in other Dock family members.

It has been found that ASD and dyslexia share similar communication deficits originated from impairments of reading or language. Indeed, difficulties of reading comprehension is a common symptoms in ASD children (prevalence ranging from 6 to 30%) (Hendren et al., 2018). Emerging evidence has suggested that both ASD and dyslexia have strong genetic components in their etiologies that involve multigene interaction (Raskind et al., 2012; Bourgeron, 2015). Notably, *DOCK4* is one of few shared candidate genes for both ASD and dyslexia. Two *DOCK4* variations have been identified in individuals with dyslexia and/or in autism subjects with poor reading abilities (Pagnamenta et al., 2010; Shao et al., 2016) (Table 1 and Figure 1A). The first variation, identified in individuals from a European family with autism and/or reading/spelling difficulties, is a microdeletion at the junction of *DOCK4* and its neighboring gene *IMMP2L* (deletion at chr7:110663978-111257682, GRCh37) (Pagnamenta et al., 2010). This variant leads to the deletion of the DHR2-containing C-terminal coding sequence of *DOCK4* (Exons 27-52), which causes a frameshift of Dock4 protein coding after 945 aa; two missense amino acids, namely Valine (Val) and Serine (Ser), is translated after 945 aa, followed by a premature stop codon (Pagnamenta et al., 2010). The second variation, identified in Chinese dyslexic children, occurs at rs20741307 and causes missense mutation of Dock4 protein, leading to a substitution of Arginine (Arg) at residue 853 with Histidine (His) (Shao et al., 2016). Previously, we showed that the protein product of the *IMMP2L*_*DOCK4* fusion transcript is unable to exert normal function on Rac1 activation and neurite outgrowth (Xiao et al., 2013). However, whether rs20741307 variation affects these Dock4 abilities has not been explored. Moreover, whether these two variations influence the synaptic regulation function of Dock4 is unclear.

**TABLE 1:** *DOCK4* variations associated with autism spectrum disorder and Dyslexia.

***DOCK4* variation^1^**	**SNP ID**	**Variant type**	**Allele change**	**Protein change**	**Protein mutant**	**Phenotype**	**Identified Population**	**References**
Deletion at 7:110663978-111257682		CNV	Exon 27-52 deleted	Frame shift after 945a.a., stop at 947a.a.	Dock4-945VS^2^	Autism and/or reading/spelling difficulties	European	Pagnamenta et al., 2010
7:111487098 (C > T)	rs2074130	Missense	c.2558G > A	p.R853H	Dock-R853H^3^	Developmental dyslexia	Chinese	Shao et al., 2016

*^1^Chromosome positions are indicated according to Assembly GRCh37. ^2^Dock4-945VS = 1–945 a.a. of Dock4 + Valine + Serine, which are two missense amino acids translated due to codon frame shift. ^3^Dock4-R853H = Dock4-Arg^853^His, in which Arginine at codon 853 is replaced by Histidine due to missense mutation at rs2074130.*

To understand the function of the two dyslexia/autism-linked *DOCK4* variations, we studied the mutant generated by the *IMMP2L*_*DOCK4* fusion transcript, Dock4-945VS (1–945 aa of Dock4 + Val + Ser), and the missense mutant of rs20741307 variation, Dock4-R853H (Arg^853^His), by examining their molecular and cellular functions through several assays. Both mutants had abrogated activities on Rac1 and Rap1 activation, and showed compromised function on promoting neurite outgrowth, synapse morphogenesis and transmission. This study provides insights in understanding the common molecular pathophysiology of autism and dyslexia by investigating their shared gene *DOCK4*.

## Materials and Methods

### Constructs, Antibodies, and Reagents

Plasmids of human Dock4 cDNA and its mutants, Dock4 945VS (amino acids 1–945 + Val + Ser), ΔSH3 (amino acids 81–1966), ΔC (amino acids 1–1592), SH3-F (amino acids 1–161), Dock shRNA, and ELMO2 were described previously (Xiao et al., 2013). cDNA of Dock4 R853H was generated by mutagenesis using the following primers, CATGTGTGCACATATCCTTAGCAACGTATT and TGCTAAGGATATGTGCACACATGATCAGGT (Sequence from 5′ to 3′). cDNA of Dock4 AAA (M1475A, S1476A, P1477A) mutant, which is deficient of GEF activity, was constructed by mutagenesis. All cDNAs with a C-terminal Flag tag were subcloned into the pCAGIG vector, which contains a GFP coding sequence separated by an internal ribosome entry site (Ip et al., 2012).

The following primary antibodies were used: Dock4, ELMO2 and GAPDH were purchased from Abcam (Cambridge, United Kingdom); α-tubulin and β-tubulin III were from Sigma; Tau1 and Rap1 were from Millipore (Darmstadt, Germany); Rac1 was from BD Biosciences; Flag was from Sigma. Retinoic acid (RA) was purchased from Sigma, and Rhodamine-phalloidin was from Invitrogen.

### Ethics Statement

All experimental procedures involving the use of animals were approved by the Ethics Committee on Animal Experiments at Jinan University, and were strictly performed according to the National Institutes of Health guidelines of the Care and Use of Laboratory Animals. All efforts were made to minimize the suffering and the number of animals used.

### *Dock4* KO Mice

*Dock4* KO mice (C57BL/6 background) were generated using a standard strategy of Cre-LoxP recombination as previously described (Guo et al., 2019).

### Cell Culture and Transfection

Neuro-2a cells and HEK293T cells were cultured as described previously (Xiao et al., 2013). COS7 cells were cultured in RPMI 1640 (Gibco) supplemented with 10% FBS. Transfection of plasmids was using Lipofectamine LTX (Invitrogen). To study neurite outgrowth, the culture medium of Neuro-2a cells was switched into differentiation medium (MEM supplemented with 0.5% FBS) in the presence of 15 μM RA for 48 h (Xiao et al., 2013; Liao et al., 2018).

Primary cultures of rat hippocampal neurons were prepared from E18 Sprague Dawley rat embryos as described previously (Xiao et al., 2013; Tang et al., 2015). Briefly, after dissection the tissue was incubated with trypsin-ETDA (0.5%, Life Technologies) in Ca^2+^ and Mg^2+^ free Hank’s balanced salt solution (HBSS with 0.06% D-Glucose, 1 mM Sodium pyruvate and 10 mM HEPES, Life Technologies) for 15 min at 37°C. Cells were washed once with plating medium (MEM supplemented with 10% horse serum, 0.6% glucose and 1 mM pyruvic acid), and suspended through trituration (Life Technologies). Cells were then plated in plating medium onto poly-D-lysine (1 mg/ml, Sigma) coated 18-mm coverslips in 12-well culture plates, at a final density of 4 × 10^4^ cells/coverslips. The plating medium was replaced with culture medium (Neurobasal medium supplemented with 2%B27 and 2 mM L-glutamine, Life Technologies) at 3 h after plating. The culture medium was half-changed every 3 days.

For analysis of hippocampal neuronal extension, plasmids were transfected into dissociated neuron suspension in Opti-MEM (Life Technologies) using Lipofectamine 2000 (Life Technologies) as previously described (Xiao et al., 2013), and neuronal morphology was analyzed at 3 days *in vitro* (DIV). For analysis of dendritic spine morphology, transfection of plasmids was performed using Calcium Phosphate precipitation at 9 DIV, and Neuronal morphology was analyzed at 16 DIV (Xiao et al., 2013).

### Immunocytochemistry

To study cell morphology and cytoskeleton in COS7 cells, cells were cultured on coverslips, transfected with indicated plasmids, and fixed in 4% paraformaldehyde (Sigma). The cells were permeabilized with a solution of 0.1% Triton X-100 in PBS for 3–5 min, and were then incubated with rhodamine-phalloidin (Invitrogen) for 20 min at room temperature. Cell morphology was photographed using a Zeiss LSM 800 confocal microscope under a 63X objective. To visualize neurites in differentiated Neuro-2a cells, cells were fixed in 4% paraformaldehyde for 20–30 min at room temperature. The cells were blocked with 1% BSA and immunostained with mouse anti-β-tubulin III antibody, followed by incubation with Alexa Fluor 546 goat anti-mouse IgG antibody (Invitrogen). Cell morphology was photographed with a Zeiss Axio Imager A2 microscope (Carl Zeiss AG, Oberkochen, Germany). Quantifications of the neurites and protrusions were made using ImageJ software (National Institutes of Health, Bethesda, MD, United States). To analyze hippocampal neuronal extension, 3 DIV neurons were fixed with 4% paraformaldehyde and subjected to immunostaining with Tau1 antibodies. Neuron morphology was photographed using a Zeiss LSM 800 confocal microscope under a 20X objective (Carl Zeiss AG). Neurite length was measured using ImageJ software. To determine dendritic spine development, 16 DIV neurons were fixed with 4% paraformaldehyde. Dendrites were photographed using a Zeiss LSM 800 confocal microscope under a 63X objective. Spine density was measured using ImageJ software.

### Electrophysiology

Whole-cell patch-clamp recordings of miniature excitatory postsynaptic currents (mEPSC) were obtained from transfected cultured hippocampal neurons at 15 DIV (Zhang et al., 2017). Briefly, cells were bathed in an external solution with a pH of 7.3 (128 mM NaCl, 5 mM KCl, 2 mM CaCl_2_, 1 mM MgCl_2_, 15 mM glucose, 20 mM HEPES, 1 mM tetrodotoxin, and 100 μM picrotoxin). Recording pipettes were filled with the intracellular solution containing (in mM): 147 KCl, 5 Na_2_-phosphocreatine, 2 EGTA, 10 HEPES, 2 MgATP, and 0.3 Na_2_GTP. Recordings were performed at room temperature in voltage clamp mode, at a holding potential of −70 mV, using a Multiclamp 700 B amplifier (Molecular Devices, Sunnyvale, CA, United States) and Clampex 10.5 software (Axon Instruments, Union City, CA, United States). The series resistance was below 30 MΩ, and data were acquired at 10 kHz and filtered at 1 kHz. mEPSCs were analyzed using MiniAnalysis software (Synaptosoft, Inc., Decatur, GA, United States).

### Rac1/Rap1 Activity Assay

Rac1/Rap1 activity was measured as previously described (Shi et al., 2007; Bai et al., 2018). Briefly, 1 day after transfection, HEK293T cells were lysed on ice in lysis buffer (25 mM Tris-HCl (pH 7.4), 1% Nonidet P-40, 2.5mM MgCl_2_, 500 mM NaCl, 10% glycerol, 10 μg/ml aprotinin and leupeptin, 1 mM PMSF). Lysates were incubated with glutathione agarose beads coupled to the p21 Rac/Cdc42 binding domain fused to GST (GST-PBD) for Rac1 activity assay, or glutathione agarose beads coupled to GST-RalGDS for Rap1 activity assay, at 4°C for 60 min with rotation. The beads were washed three times with lysis buffer and resuspended with sample buffer. Samples were subjected to Western blot analysis. Signal intensity was quantified by densitometry using ImageJ software.

### Statistical Analysis

Data are represented as means ± SEM and were analyzed with Prism 7.0 (GraphPad Software). Comparisons between two experimental groups were performed with unpaired Student’s *t-*test. Comparisons among three or more groups were performed with one-way ANOVA. Differences were considered significant if *P* < 0.05.

## Results

### Arg^853^ Is a Unique Site of Dock4 Among All Dock Families and Is Highly Conserved During Evolution

Arg^853^ of human Dock4 is located in the linker region between DHR1 and DHR2 domains (Figure 1A), a region that has no known molecular function yet. We searched the UniProtKB Protein knowledgebase^1^ and compared the sequence of amino acid sequences of Dock4 among different species, including frog (*Xenopus tropicalis*), mouse (*Mus musculus*), rat (*Rattus norvegicus*), marmoset (*Callithrix jacchus*), rhesus macaque (*Macaca mulatta*), chimpanzee (*Pan troglodytes*), and human (*Homo sapiens*). By using the Multiple Sequence Alignment analysis tool of Clustal Omega^2^, we found that the Arg^853^ and its flanking sequences are highly conserved during evolution (Figure 1B). However, this region is not conserved in other Dock family members (Figure 1C), suggesting that this region is unique in Dock4.

### Both Dock4-R853H and 945VS Lose the GEF Activities to Activate Rac1 and Rap1 GTPases

**FIGURE 2:**
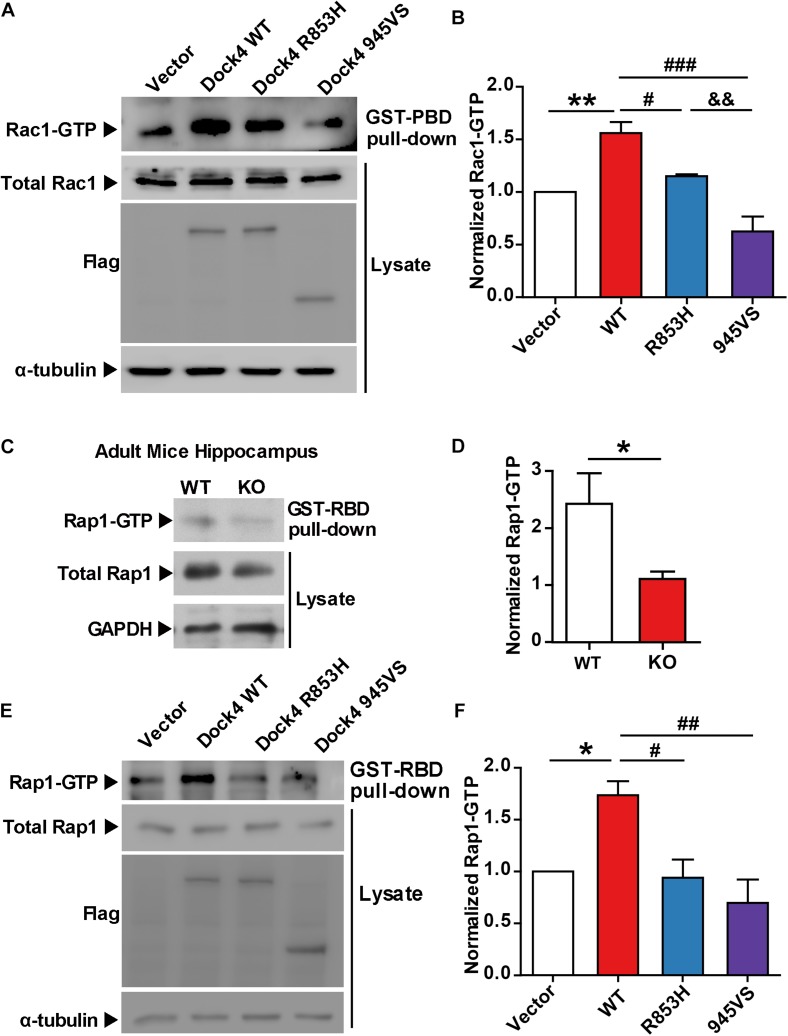
Both 945VS and R853H mutants of Dock4 lose the GEF activities to activate Rac1 and Rap1 GTPases. **(A)** Rac1 activation by Dock4 mutants was abrogated. cDNAs of Dock4-R853H, Dock4-945VS, Dock4-WT (wild-type), or vector were expressed in HEK293T cells, and the levels of activated Rac1 (Rac1-GTP) were measured. **(B)** Rac1-GTP levels were quantified and normalized to total Rac1 levels. Data are shown as mean ± SEM from three independent experiments. ^∗∗^*P* < 0.01, #*P* < 0.05, ###*P* < 0.001, &&*P* < 0.01, one-way ANOVA. **(C)** Rap1-GTP level was reduced in hippocampus of *Dock4* knockout mice. Adult WT and *Dock4* knockout hippocampus protein was lysed, and the levels of Rap1-GTP were analyzed. **(D)** Rap1-GTP levels were quantified and normalized. Data are shown as mean ± SEM from three independent experiments. ^∗^*P* < 0.05, unpaired *t*-test. **(E)** Dock4 mutants failed to activate Rap1. Dock4-R853H, Dock4-R853H or Dock4-WT were expressed in HEK293T cells, and the levels of Rap1-GTP were analyzed. **(F)** Rap1-GTP levels were quantified and normalized. Data are shown as mean ± SEM from three independent experiments. ^∗^*P* < 0.05, #*P* < 0.05, ##*P* < 0.01, one-way ANOVA.

To study whether the autism/dyslexia-linked variations of *DOCK4* have influences on its Rac1 GEF activity, we compared the abilities of Rac1 activation by Dock4-945VS and R853H with that of the wild-type (WT) protein when exogenously expressed in cells. As previously reported (Xiao et al., 2013), 945VS totally lost the Rac1-activation ability (Figures 2A,B). R853H also decreased activated-Rac1 levels when compared to WT, although the reduction was not as robust as that caused by 945VS (Figures 2A,B). Besides the ability to activating Rac1, Dock4 is also known to have Rap1 GEF activity in tumor cells (Yajnik et al., 2003). We showed here that Rap1 activation was significantly compromised in *Dock4* KO hippocampus (Figures 2C,D), suggesting that Dock4 is capable of activating Rap1 in nervous system. We then examined the abilities of exogenously expressed Dock4-945VS and R853H toward Rap1 activation. Notably, while Dock4-WT remarkably increased activated-Rap1 levels, both mutants lacked this ability (Figures 2E,F). Hence, the two autism/dyslexia-linked mutants of Dock4 showed impaired abilities on activation of both Rac1 and Rap1.

### The SH3 Domain and DHR2 Domain Are Important for Dock4-Regulated Rap1 Activation

**FIGURE 3:**
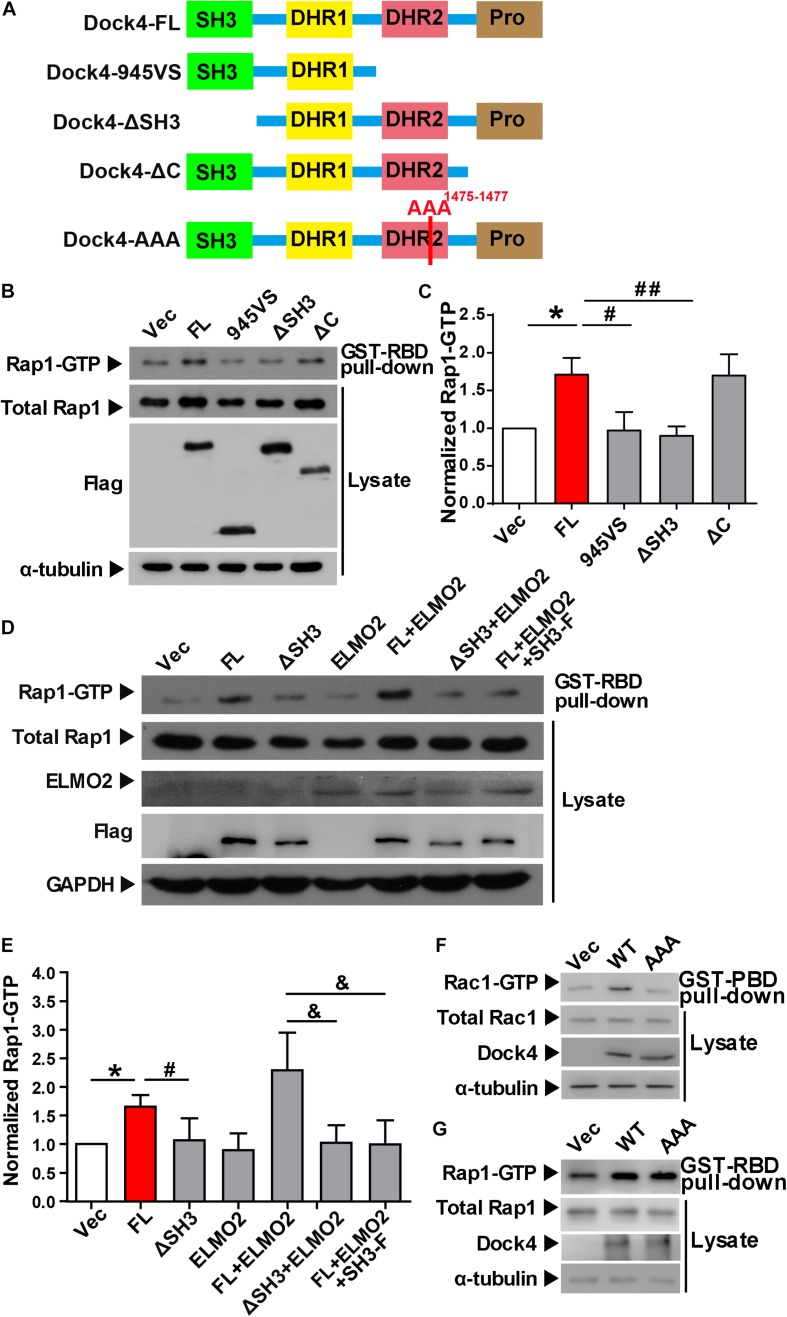
The SH3 domain and DHR2 domain are important for Dock4-regulated Rap1 activation. **(A)** Domain illustrations of Dock4 and its mutants. **(B)** Rap1 activation was analyzed after Dock4-FL and various deletion mutants were transfected in HEK293T cells. **(C)** Rap1-GTP levels were quantified and normalized to total Rap1 expression. Data are shown as mean ± SEM from three independent experiments. ^∗^*P* < 0.05, #*P* < 0.05, ##*P* < 0.01, one-way ANOVA. **(D)** Dock4 activates Rap1 through SH3 domain coupling with ELMO2. ELMO2 and Flag-tagged Dock4-FL, ΔSH3 or SH3-F were co-transfected into HEK293T cells as indicated, and the levels of Rap1-GTP were analyzed. **(E)** Rap1-GTP levels were quantified and normalized. Data are shown as mean ± SEM from three independent experiments. ^∗^*P* < 0.05, #*P* < 0.05, &*P* < 0.05, one-way ANOVA. **(F,G)** The GEF-dead Dock4-AAA mutant lost Rac1-activating ability, but had intact Rap1-activating ability. Dock4-FL and its AAA mutant were transfected in HEK293T cells. Rac1-GTP **(F)** or Rap1-GTP **(G)** levels were examined.

Previously, we found that both the SH3 domain and the DHR2 domain, but not the C terminus, are required for Dock4 GEF activity toward Rac1 (Xiao et al., 2013). We went on to study whether these domains are important for activation of Rap1. Similarly, we observed that the SH3-lacking Dock4 (ΔSH3) could not activate Rap1, whereas the C-terminus-lacking Dock4 (ΔC) had intact Rap1-activation ability (Figures 3A–C). As 945VS, which lacks both DHR2 and the C-terminus, lost the Rap1-activating ability, it suggests that the DHR2 is responsible for not only Rac1, but also Rap1 activation. It has been shown that the Dock4 SH3 domain interacts with ELMO2 (Engulfment and cell motility 2), which acts as a co-factor of Dock4 toward Rac1-activation (Patel et al., 2011; Xiao et al., 2013; Laurin and Cote, 2014). To study whether ELMO2 is also important for Rap1 activation, we expressed ELMO2 together with Dock4 full length (FL) or ΔSH3 protein. Notably, ELMO2, without altering Rap1 levels by itself, appeared to further substantiate the effect of Dock4-FL on Rap1 activation (Figures 3D,E). In contrast, ELMO2 did not have any effect on Rap1 in Dock4-ΔSH3-expressing cells (Figures 3D,E). We further used a fragment of N-terminal Dock4 containing the SH3 domain (SH3-F) to compete the ELMO2 binding with Dock4-FL (Xiao et al., 2013). Consistently, when SH3-F was added to the Dock4-FL and ELMO2 complex, the Rap1 activation was completely abolished (Figures 3D,E). These results suggest that ELMO2, through forming a complex with Dock4, promotes the function of Dock4’s DHR2 domain on both Rac1 and Rap1 activation. As the DHR2 of Dock4 is known as a RacGEF domain, we asked whether its RacGEF catalytic activity is responsible for Rap1 activation. To this end, a RacGEF-dead mutant of Dock4 (in which M1475, S1476, and P1477 in the DHR2 domain are all mutated to alanine), the AAA mutant, was examined (Figure 3A). Interestingly, the AAA mutant did lose the ability to activate Rac1, but its Rap1-activating ability was unaltered (Figures 3F,G). Therefore, Dock4 does not activate Rap1 the same way as it activates Rac1, and alternate mechanisms besides RacGEF activity may underlie the requirement of DHR2 domain for Rap1 activation.

### Dock4-R853H and 945VS Fail to Influence Cell Morphology

**FIGURE 4:**
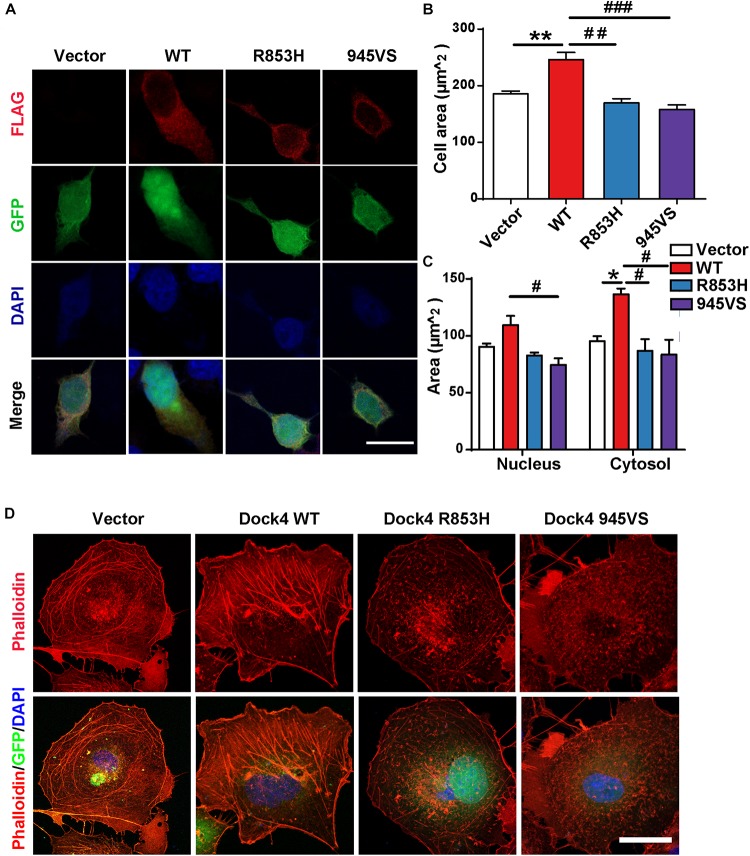
Dock4-R853H and 945VS fail to influence cell morphology. **(A)** Dock4-R853H, Dock4-945VS, Dock4-WT or vector have different morphology in HEK293T. We transfected HEK293T cells with Vector, Flag-tagged Dock4-WT, Dock4-R853H, Dock4-945VS plasmids. Cells were immunostained using Flag antibody. Scale bar, 20 μm. **(B,C)** Total cell area, cytoplasmic area, and nuclear area were measured. Data are shown as mean ± SEM from three independent experiments. ^∗^*P* < 0.05, ^∗∗^*P* < 0.01, #*P* < 0.05, ##*P* < 0.01, ###*P* < 0.001, one-way ANOVA. At least 20 cells/group were analyzed in each experiment. **(D)** Dock4 R853H and 945VS disrupt actin cytoskeleton in COS7 cells. Cells were immunostained using Phalloidin for visualization of actin cytoskeleton. Scale bar, 50 μm.

We have shown that Dock4-R853H and 945VS exhibit decreased activities toward both Rac1 and Rap1, which regulate cell morphology by influencing cytoskeleton and cell adhesion. Indeed, HEK293T cells overexpressed with Dock4-WT exhibited a spread, elongated morphology with enlargement of both cytosolic and nuclear regions when compared to the vector-expressed cells (Figures 4A–C). The cellular location of Dock4-R853H and 945VS was mainly enriched in the cytoplasm, which was similar to that of Dock4-WT (Figure 4A). However, Dock4-R853H and 945VS failed to influence the overall cell morphology, as the cellular size was not changed by either mutant (Figures 4A–C). We then examined the cytoskeleton in cells expressing different Dock4 forms. Interestingly, strong staining of F-actin was observed in the cytoplasm and at the membrane of cells expressed with Dock4-WT (Figure 4C). In contrast, much weaker F-actin staining was observed in R853H- or 945VS-expressed cells (Figure 4D). Therefore, Dock4-R853H and 945VS are dysfunctional to regulate cell morphology and cytoskeleton properly.

### Dock4-R853H and 945VS Lack Neurite Outgrowth Abilities

**FIGURE 5:**
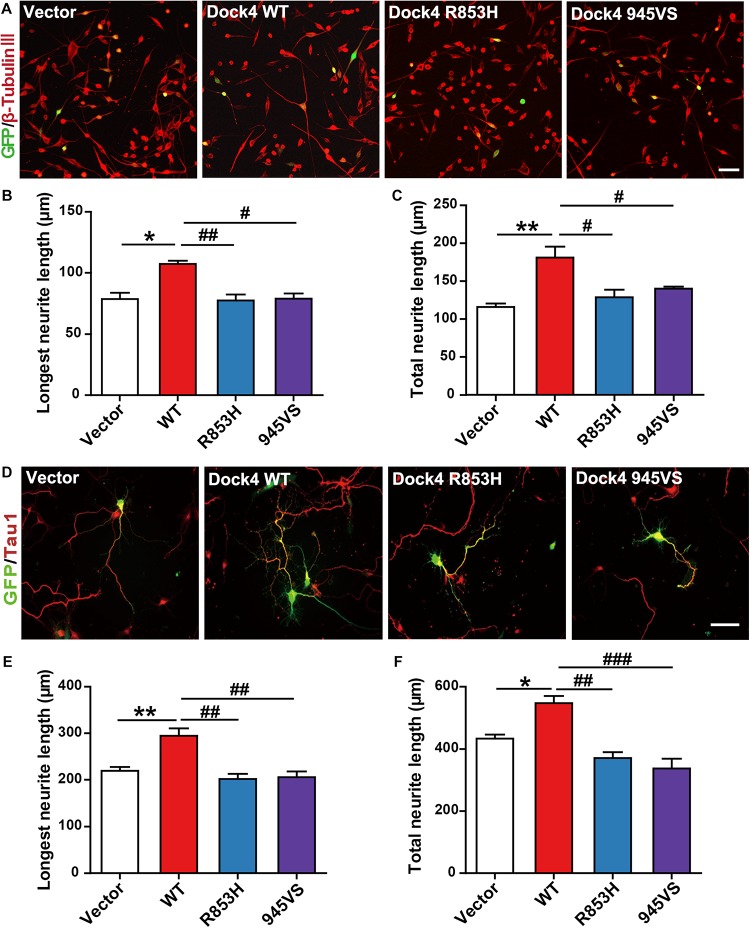
Dock4-R853H and 945VS lack neurite outgrowth abilities. **(A)** Dock4 mutants could not promote RA-induced neurite outgrowth in Neuro-2a cells. Neuro-2a cells were transfected with plasmids expressing Dock4-WT, Dock4-R853H, Dock4-945VS or Vector, followed by treatment with RA (15 μM) for 48 h. Cells were immunostained using β-tubulin III antibody for visualization of neurites, and transfected cells were indicated by GFP. Scale bar, 100 μm. **(B,C)** Average length of the longest neurite and average length of total neurites were measured. ^∗^*P* < 0.05, ^∗∗^*P* < 0.01, #*P* < 0.05, ##*P* < 0.01, one-way ANOVA. At least 40 cells/group were analyzed in each experiment. **(D)** Dock4 mutants could not promote neurite outgrowth in hippocampal neurons. Dissociated E18 hippocampal neurons were transfected with plasmids expressing Dock4-WT, Dock4-R853H, Dock4-945VS or Vector, cultured for 3 days. Axon-like process were immunostained with Tau1 (red) antibody, and transfected cells were indicated by GFP. Scale bar, 50 μm. **(E,F)** Average length of the longest neurite and average length of total neurites were measured. ^∗^*P* < 0.05, ^∗∗^*P* < 0.01, #*P* < 0.05, ##*P* < 0.01, ###*P* < 0.001, one-way ANOVA. At least 40 cells/group were analyzed in each experiment.

Previous evidence has revealed that Dock4 is important for neurite outgrowth and synapse morphogenesis during neuronal development (Ueda et al., 2008, 2013; Xiao et al., 2013). We first examined the ability of Dock4 mutants on promoting neurite outgrowth in Neuro-2a cells, a neuron-like cell line widely used for studying neuronal differentiation and neurite extension. When transfected into Neuro-2a cells, Dock4 substantially promoted neurite elongation upon retinoic acid (RA) stimulation (Figures 5A–C). However, neither Dock4-R853H nor Dock4-945VS was able to influence neurite outgrowth, as the neurite length of cells expressed with either mutant was comparable to that of the vector-expressed cells (Figures 5A–C). We further studied the neurite regulating roles of the two Dock4 mutants in cultured hippocampal neurons. We transfected either mutant in dissociated neurons before plating, and examined the neurite morphology at 3 DIV, by which time extensive neurites are formed with one differentiated into the axon. As observed in Neuro-2a cells, both mutants lost the ability to promote axon or total neurite elongation when compared to the WT protein (Figures 5D–F).

### Dock4-R853H and 945VS Have Compromised Abilities to Promote Dendritic Spine Morphogenesis and Synaptic Transmission

**FIGURE 6:**
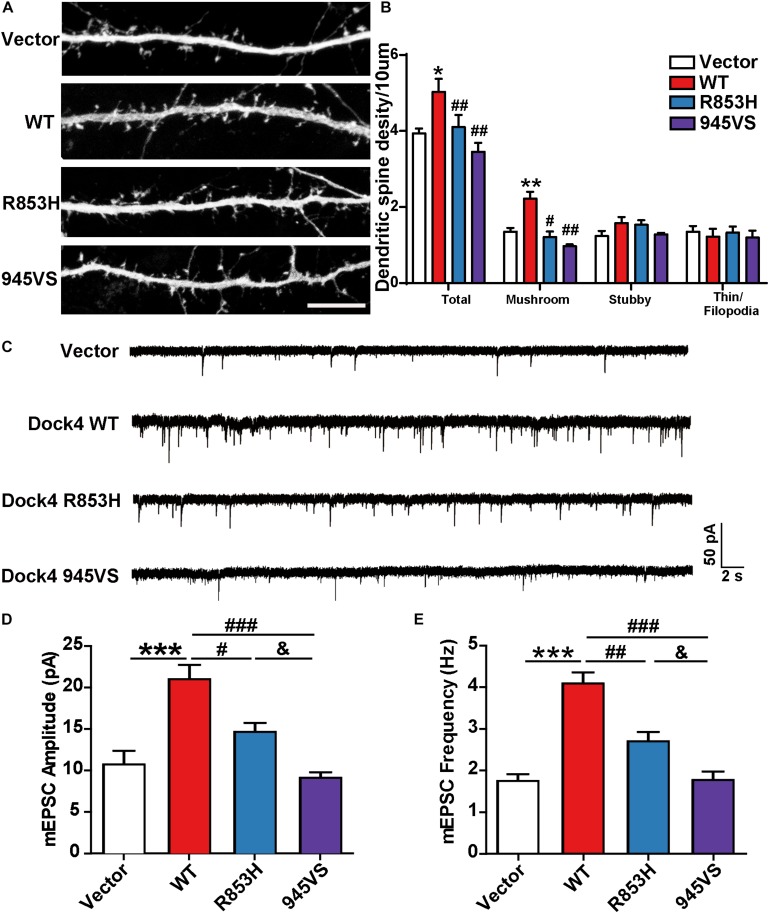
Dock4-R853H and 945VS have compromised abilities to promote dendritic spine morphogenesis and synaptic transmission. **(A)** Dock4 mutants could not promote dendritic spine formation in hippocampal neurons. 9 DIV hippocampal neurons were transfected with plasmids expressing Dock4-WT, Dock4-R853H, Dock4-945VS or Vector and analyzed at 16 DIV. Scale bar, 10 μm. **(B)** Density of total spines and different types of spines were measured. Data are shown as mean ± SEM from three independent experiments. ^∗^*P* < 0.05, ^∗∗^*P* < 0.01, #*P* < 0.05, ##*P* < 0.01, one-way ANOVA. At least 30 cells/group were analyzed in each experiment. **(C)** Miniature excitatory postsynaptic currents (mEPSCs) of hippocampal neurons expressed with Dock4-WT, Dock4-R853H, Dock4-945VS or Vector. Amplitude **(D)** and frequency **(E)** were quantified. ^∗∗∗^*P* < 0.001, #*P* < 0.05, ##*P* < 0.01, ###*P* < 0.001, &*P* < 0.05, one-way ANOVA. At least 10 cells/group were analyzed in each experiment.

Previous studies in both *Dock4* KO mice and in Dock4-shRNA knockdown neurons have shown that Dock4 promotes dendritic spine formation and excitatory synaptic transmission in hippocampus (Ueda et al., 2013; Guo et al., 2019). To investigate the abilities of Dock4-R853H and 945VS on spine morphogenesis, we delivered each plasmid into cultured hippocampal neurons by calcium phosphate transfection at 9 DIV, and the co-expressed GFP was used to indicate morphology of the transfected neurons. The spine number was examined at 16 DIV. As reported before, we observed a remarkable increase of spine density in Dock4-WT expressed neurons as compared to vector expressed neurons (Figures 6A,B). However, neither R853H nor 945VS was able to influence spine density (Figures 6A,B). By quantifying different types of spines, we found that Dock4 mainly promoted the mushroom-shaped spine, which is believed as the mature spines, whereas both mutants lacked this ability (Figure 6B). To further examine synaptic transmission, we measured miniature excitatory postsynaptic current (mEPSC) in these neurons by electrophysiological recordings (Figure 6C). Consistently, Dock4-WT increased both amplitude and frequency of mEPSC, suggesting that there were more functional excitatory synapses in Dock4 WT expressed neurons than in vector expressed neurons (Figures 6C–E). Interestingly, Dock4-R853H also increased mEPSC amplitude and frequency, but the effect was significantly weaker than that of Dock4-WT (Figures 6C–E). By contrast, Dock4-945VS completely lost the ability to promote mEPSC (Figures 6C–E). Together, these findings suggest that Dock4-R853H retains partial function, whereas 945VS loses the whole function on regulating excitatory synaptic transmission.

### Rac1 and Rap1 Rescue the Defects of Neurite Outgrowth and Spine Morphogenesis in Dock4 Knockdown Neurons

**FIGURE 7:**
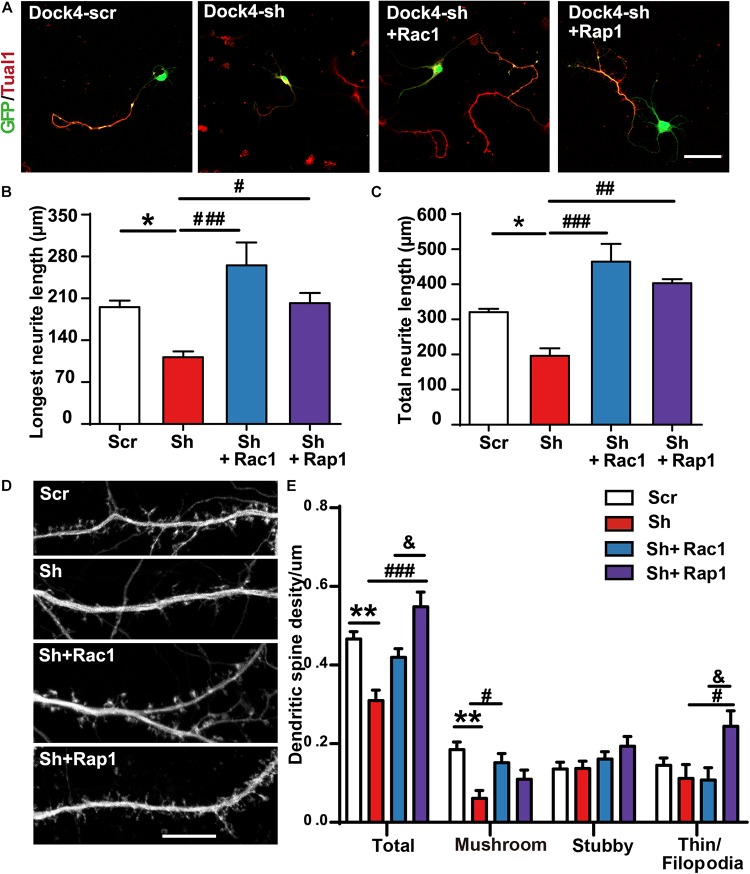
Rac1 and Rap1 rescue the defects of neurite outgrowth and spine morphogenesis in Dock4 knockdown neurons. **(A)** Rac1 and Rap1 rescue neurite outgrowth defects in Dock4-knockdown hippocampal neurons. Dissociated E18 hippocampal neurons were transfected with plasmids expressing Dock4-scramble shRNA (Scr), Dock4-shRNA (sh), sh + Rac1, or sh + Rap1, and cultured for 3 days. Axon-like processes were immunostained with Tau1 (red) antibody, and transfected cells were indicated by GFP. Scale bar, 50 μm. Average length of the longest neurite **(B)** and total neurites **(C)** was measured. ^∗^*P* < 0.05, #*P* < 0.05, ##*P* < 0.01, ###*P* < 0.001, one-way ANOVA. At least 40 cells/group were analyzed in each experiment. **(D)** 9 DIV hippocampal neurons were transfected with plasmids expressing Scr, sh, sh + Rac1, or sh + Rap1. Spine morphology was analyzed at 16 DIV. Scale bar, 10 μm. **(E)** Densities of total spines and different types of spines were measured. Data are shown as mean ± SEM from three independent experiments. ^∗∗^*P* < 0.01, #*P* < 0.05, ###*P* < 0.001, &*P* < 0.05, one-way ANOVA. At least 20 cells/group were analyzed in each experiment.

To investigate whether Rac1 and Rap1 differentially participate in Dock4 function, we overexpressed either Rac1 or Rap1 in Dock4 knockdown hippocampal neurons. We studied the rescue effect of Rac1 or Rap1 in two stages of neuronal development. First, transfection of Dock4 shRNA together with either Rac1 or Rap1 was performed before neuronal cell plating, and neurite morphology was examined at 3 DIV (Figure 7A). Whereas Dock4 shRNA decreased neurite length significantly, co-expression of either Rac1 or Rap1 was able to rescue this impaired neurite outgrowth (Figures 7B,C). Notably, the effect of Rac1 appeared to be stronger than Rap1 (Figures 7B,C). Second, transfection of Dock4 shRNA together with either Rac1 or Rap1 was performed at 9 DIV, and spine morphology was examined at 16 DIV (Figure 7D). As reported before (Ueda et al., 2013), knockdown of Dock4 resulted in dramatic decrease of spine density, especially the density of the mushroom-shaped mature spines (Figures 7D,E). Importantly, overexpression of Rac1 specifically restored the density of these mature spines in Dock4-knockdown neurons (Figures 7D,E). Overexpression of Rap1, on the other hand, only promoted growth of filopodia/thin type of spines which are believed as the dynamic immature spines, but could not reverse the decrease of mature spines in Dock4-knockdown neurons (Figures 7D,E). These observations are consistent with the current knowledge on Rac1 and Rap1, which are important for spine enlargement and dynamics, respectively (Woolfrey and Srivastava, 2016). Together, the rescue experiments at different neuronal developmental stages suggest that Rac1 and Rap1 may corporately participate in Dock4-mediated neurite outgrowth, and Rac1 is specifically required for Dock4-mediated formation of mature spines.

## Discussion

ASD and dyslexia are both neurodevelopmental disorders with high prevalence in children. As there are not many comorbidity studies of ASD and dyslexia, it is arguable whether the co-occurring reading difficulties in ASD are the same as in dyslexia, which is mainly characterized by phonological deficits (Hendren et al., 2018). Nonetheless, both diseases are well-documented comorbid diseases of language impairment (LI) with similar language traits, suggesting that ASD may share etiologies of language and verbal communication deficits with dyslexia and LI (Eicher and Gruen, 2015). Despite heterogeneous origins, emerging genetic and pathological findings point to a conclusion that ASD is a disease with impaired neuronal connectivity (Bourgeron, 2015). A large amount of high risk ASD genes play important roles at the synapse, in particular, at the postsynaptic sites of the excitatory synapses. These include synaptic receptors, adhesion molecules, scaffolding proteins and cytoskeletal regulators. The molecular pathophysiology of dyslexia is less understood, but lines of evidence suggest that disruptions of neuronal migration and neurite outgrowth contribute importantly to the pathogenesis of the disease (Poelmans et al., 2011; Shao et al., 2016; Guidi et al., 2018). So far, very few shared risk genes have been identified in both ASD and dyslexia. As a candidate gene for both disorders, *DOCK4* draws attention for understanding their common etiology.

The current study focuses on two *DOCK4* variants, Exon27-52 deletion (protein product: Dock4-945VS and rs2074130 variation (protein product: Dock4-R853H), which are associated with dyslexia and/or ASD with reading difficulties. Among them, the molecular function of Dock4-R853H is uncharacterized before. In the initial biochemical assays, we found that the RacGEF activities of both mutants are compromised. The loss of RacGEF activity is expected for Dock4-945VS because this mutant lacks the entire GEF domain, i.e., DHR2 domain. Interestingly, R853H also showed moderate decrease in RacGEF activity. The current study further confirms that Rap1 is another small GTPase besides Rac1 that can be activated by Dock4 in brain. Both Dock4-945VS and R853H totally lost the ability to activate Rap1. Domain mapping analysis suggests that similar to Rac1 activation (Xiao et al., 2013), DHR2 domain as well as binding to ELMO2 via SH3 domain are necessary for Rap1 activation. However, the RacGEF catalytic activity of DHR2 is not necessary for Rap1 activation, suggesting Dock4 uses different mechanism to activate Rac1 and Rap1 respectively. Further studies will be required to investigate whether DHR2 directly activates Rap1 or via binding to other Rap1-regulating proteins. Nonetheless, these studies suggest that R853 is probably a critical residue for the regulation of the DHR2 domain function. R853 and its flanking regions are unique sequence in Dock4 among Dock family members, it may represent a special site of regulation only in Dock4 but not in other Docks. Further analysis will be necessary to understand whether this residue is important for directly maintaining the structural integrity of the protein, or for recruiting other critical regulators that in turn control the function of the protein.

Cellular assays in this study reveal that both Dock4-R853H and 945VS lack the abilities to influence cell morphology, promote neurite outgrowth, and induce spine morphogenesis. By using electrophysiological approaches, we further demonstrate that Dock4-R853H is still able to promote excitatory synaptic transmission, although this ability is weaker than the WT protein. Dock4-945VS, on the other hand, completely fails to regulate synaptic transmission. This difference could be due to the fact that Dock4-R853 still retains some level of Rac1-activating ability, whereas 945VS lost both Rac1- and Rap1-activating abilities. Indeed, we showed that Rac1 is mainly required for the formation of mature spines regulated by Dock4, suggesting that Rac1 may be the primary small GTPases downstream of Dock4 for functional synapse formation and transmission.

Findings in this study are consistent with the neurodevelopment impairment hypothesis of ASD and dyslexia, in which *DOCK4* contributes to a shared genetic factor for both disorders. Using *Dock4* knockout mice, we have shown that deficiency of *Dock4 in vivo* leads to social and communication deficits (Guo et al., 2019). Findings of the current study provide further molecular and cellular evidence that Dock4-dependent regulation of neuronal development may underlie the neurobiology basis of social communication and language development. Therefore, investigating the mechanism by which Dock4 and its downstream molecules regulate neuronal function and behavior provides more evidence for understanding the pathogenesis of the co-occurring symptoms of ASD and dyslexia.

## Data Availability Statement

All datasets generated for this study are included in the article/supplementary material.

## Ethics Statement

The animal study was reviewed and approved by the Jinan University.

## Author Contributions

MH, CL, SL, JZ, DG, BZ, and YP performed the experiments. YL and WL purified the Rac1- and Rap1-binding protein domains for Rac1 and Rap1 assays. MH, CL, SL, YP, GG, and LS analyzed the data. MH, CL, SL, JX, and LS did data interpretation and figure organization. MH and LS wrote the manuscript.

## Conflict of Interest

The authors declare that the research was conducted in the absence of any commercial or financial relationships that could be construed as a potential conflict of interest.

## Footnotes

1 http://www.uniprot.org/

2 http://www.ebi.ac.uk/Tools/msa/clustalo/
